# Quality of Life in Palliative Care: A Systematic Meta-Review of Reviews and Meta-Analyses

**DOI:** 10.2174/0117450179183857240226094258

**Published:** 2024-03-05

**Authors:** Mauro Demuro, Elisa Bratzu, Stefano Lorrai, Antonio Preti

**Affiliations:** 1 Department of Medical Sciences and Public Health, University of Cagliari, Cagliari, Italy; 2 Department of Neuroscience “Rita Levi Montalcini”, University of Turin, Turin, Italy

**Keywords:** Quality of life, Palliative care, Cancer, Advanced illness, Advanced heart failure, Meta-analysis

## Abstract

**Background:**

The area of palliative care is a setting in which the evaluation of the quality of life (QoL) is fundamental. However, the topic has been covered from many different points of view, and there is a lack of comprehensive synthesis of the evidence drawn from the available literature.

**Objective:**

We carried out a meta-review of all available systematic reviews and meta-analyses that have dedicated part or most of the investigation to the assessment of QoL in palliative care to provide the most updated and comprehensive depiction of all available information about measurement and intervention aimed at improving QoL in palliative care.

**Methods:**

A meta-review of all recent (5 years) available systematic reviews and meta-analyses on “palliative care” and “quality of life” was carried out. The quality of the extracted studies was assessed with the AMSTAR scale.

**Results:**

The search extracted 24 systematic reviews, 14 systematic reviews followed by a meta-analysis on a subset of data, and 2 meta-analyses. In many studies, the investigation of QoL represented a secondary or even marginal outcome. In general, the results supported the efficacy of palliative care in terminal patients or patients with a permanent disability. However, the quality of the studies had a strong influence on the chance that some improvement in QoL was found in relation to palliative care. Studies of lower quality were more likely to report some efficacy of palliative care than studies with better quality.

**Conclusion:**

The investigation of QoL in palliative care is understudied. In many studies, QoL is a secondary outcome, and there is some tendency to use a disparate range of tools to measure it, whose reliability and validity should still be established in some groups of patients.

## 1 INTRODUCTION

The World Health Organization Quality of Life (WHOQOL) group in 1995 defined quality of life (QoL) as “the perception that individuals have of their position in life in the context of the culture and value systems in which they live and in relation to their goals, expectations, standards, and concerns”. Six fundamental domains or key aspects of QoL were proposed: physical well-being, psychological well-being, level of independence, social relationships, environment, personal beliefs, and/or spirituality. However, so far, the scientific community still struggles to find a concordant definition, as the concept of QoL is wide and subjective. Some believe in a limited set of concepts: QoL is a subjective construct, or it must be evaluated by the subject. It is multidimensional and dynamic; thus, it may change based on time and circumstances, and finally, it is related to the culture of the subject. Despite some inconsistencies, the construct of QoL is important for clinical purposes. The QoL assessment allows us to quantify the impact of the patients’ clinical condition and their possible treatment on the most varied aspects of their life.

Nevertheless, the evaluation of QoL is a complex subject, which includes a great heterogeneity of tools with considerable methodological and statistical difficulties. General purposes tools and specific measures were developed to tailor QoL in selected samples of patients, such as those with cancer or chronic diseases. A detailed description of the tools used in the studies that were reviewed in the present meta-review is reported further in the given Table.

The area of palliative care is a setting in which the evaluation of the QoL is fundamental. The WHO defines palliative care as an approach capable of improving “the quality of life of patients and their families, who are faced with the problems associated with incurable diseases, through prevention and relief of suffering through early identification and optimal treatment of pain and other problems of a physical, psychosocial, and spiritual nature” (World Health Organization, National Cancer Control Programs. Policies And Managerial Guidelines, 2002, p. 84) [1]. The term “palliative” indicates that the intervention does not act on the cause of the disease (etiological therapy) but acts to relieve refractory symptoms, therefore the suffering and the problems related to the pathology. There is a wide and varied dissemination of studies about QoL in palliative care. However, the topic has been covered from many different points of view, and there is a lack of comprehensive synthesis of the evidence drawn from the available literature. Moreover, a still-controversial topic concerns the role of Health-Related QoL (HRQoL) in terminal or permanently disabling diseases, which are among the major causes of discomfort and poor QoL and are often treated in palliative care when the etiologic cure is no more effective.

We carried out a meta-review of all available systematic reviews and meta-analyses that have dedicated part or most of the investigation to the assessment of HRQoL in palliative care with special reference to the most widespread terminal or permanently disabling diseases. The main goal of this meta-review is to provide the most updated and comprehensive depiction of all available information about measurement and intervention aimed at improving the HRQoL in palliative care.

## 2 METHODS

This systematic review was conducted according to the Preferred Reporting Items for Systematic Reviews and Meta-Analyses (PRISMA) [2]. Available literature was explored with a search in PubMed/Medline (https://pubmed.ncbi.nlm.nih.gov/) and the Cochrane Library (https://www.cochranelibrary.com/). The most recent articles were searched, with an interval from 1^st^ January, 2015 to 31^st^ December, 2020. The simplest combination of keywords was used to increase the comprehensiveness of the search: “palliative care” and “quality of life”. Articles were included when they were written in English, were published in peer-review journals, and detailed the results of a systematic review or a meta-analysis. No limitation on age, sex, religion, or geographic origin was applied to the search. Three independent researchers (MD, EB, and SL) cross-checked the reports found in the search results, checking for the title, abstract, and text to confirm their eligibility. Each step of inclusion/exclusion was supervised by a fourth experienced researcher (AP).

**Fig. (1):**
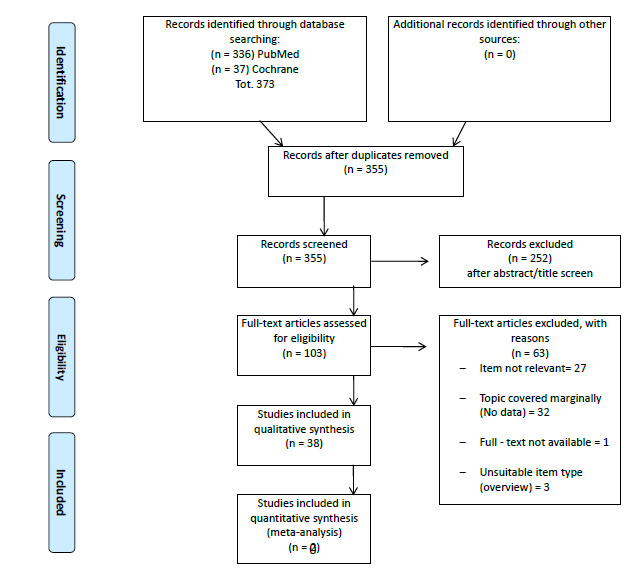
PRISMA flowchart of studies reporting data on quality of life in patients admitted to palliative care.

The search retrieved 373 articles; 18 articles were excluded as duplicates. From the remaining 355 articles, 252 articles were eliminated based on their title/abstract since they were not a systematic review or a meta-analysis or were not congruent with the topic of the search. The remaining eligible 103 were inspected in full text; a total of 63 were excluded because they were not relevant to the research, the topic was mentioned only marginally, the full text was not available, or they were not articles. Overall, 40 articles were found to be suitable for qualitative assessment and data extraction (Fig. **1**).

The extracted articles were inspected for references of each review suitable for inclusion, and the procedure was repeated for each new potential review. No suitable additional reference was found.

From each article, data of interest were extracted by three investigators under the supervision of an independent fourth investigator. The extracted data were organized in a table in chronological order by setting every single article from the oldest to the most recent and grouping the information into the following fields:

1: Type of study (systematic review or meta-analysis),

2: Geographic location of the studies included in the systematic review or meta-analysis,

3: Characteristics of the included samples,

4: Medical condition under investigation,

5: Diagnosis,

6: Evaluation tools for the assessment of QoL and

7: Main findings.

Table **1** lists the details concerning the main charac-teristics and findings of the included systematic reviews and meta-analyses.

The potential risk of bias was assessed with the AMSTAR scale [3, 4]. For this study, the articles were evaluated according to the following rule: any “yes” was scored 1; instances of “no” or “cannot say” were scored 0. The articles that received a global score of 1 to 6, 7 to 9 and 10 to 11 were evaluated, respectively, as “Unaccep-table,” “Acceptable,” and “High Quality” articles. Articles that received a score of 0 were rated as “Rejected” and consequently excluded from the search, just like articles that scored 1 to 6 (Unacceptable) (Table **2**).

## 3 RESULTS

The included 40 articles were: 23 systematic reviews [5-27], 15 systematic reviews completed with a meta-analysis [28-41], and 2 meta-analyses [42, 43].

### 3.1 Sample Size and Characteristics of the Included Studies

The results obtained relating to QoL were often extrapolated from the selected articles since not all research had QoL as the main topic. For this reason, we carefully analyzed each search to minimize the possibility of entering incorrect data in the results. The largest sample size was found in a study by Diop *et al*. [32], with 24,403 participants, while the smaller one was found in a study by Abdel-Rahman *et al*. [21]. It should be noted that a few articles reported no sample size information or data (Table **1**).

Regarding geolocation, the articles included results from different countries; most studies were conducted in North America, Europe, and China and less frequently in Central and South America, Australia, South Africa, Israel, Japan, and the former Soviet Union.

### 3.2 Evaluation Tools for the Assessment of the QoL

Most articles reported the evaluation tools that were used to assess QoL (Table **3**).

The most frequently assessed dimensions were the mental, physical, and functional health status, while the measurement tools only aimed at the assessment of the physical health status were rarely used, and many studies did not report QoL results about this dimension. Several tools were also found for measuring QoL in patients with specific pathologies, tools for measuring QoL in pediatric patients, and tools for measuring QoL of families or staff caring for the patient in palliative care or in the hospital.

The most commonly administered questionnaires were the EORTC-QLQ (various versions), SF-36, MQOL, and EUROQOL EQ-5D. Several studies did not directly report the measurement tools used but only the data.

### 3.3 Quality of the Assessment Tools and Risk of Bias

It was found that there were problems in the reliability of the results due to the high mortality of patients, often due to pathology or other complications, and therefore, in many studies, there was a lack of follow-up. This problem, leading to the loss of critical information, was found in most articles dealing with terminal illnesses, and many of these studies reported the data as “statistically significant but not clinically relevant” due to the risk of bias.

### 3.4 Disorders and Pathologies

In the included articles, we found various pathologies and carefully selected those that exclusively concerned the possibility of receiving treatments that include palliative care. Of these, we distinguished 2 categories, terminal pathologies and permanently disabling pathologies (both respect the standards of the study of QoL in palliative care). The first group included tumors, cardiac arrest, HIV / AIDS, and a combination of them (multiple tumors, cardiac arrest associated with tumors, *etc*.). The chronic diseases included multiple sclerosis, chronic obstructive pulmonary disease, Parkinson's disease, chronic pruritus, hemodialysis, dyspnea, and chronic kidney disease.

### 3.5 Treatments

Many treatments have been proposed in the studies, all related to palliative care.

Of these, 5 used a placebo in the control group [9, 10, 12, 14, 33], 4 had a control group without palliative care [6, 8, 17, 32], and the remaining ones had specific therapies *versus* the lack of them in the control.

Among the prescribed therapies, there were:

#### 3.5.1 Protocols Based on the Administration of Drugs or Integrators

- Administration of vitamins,

- Administration of minerals,

- Administration of proteins,

- Administration of morphine or other opioids,

- Checkpoint inhibitor therapy.

#### 3.5.2 Protocols Based on Psychosocial Interventions

- Psychotherapy,

- Music therapy,

- Acupuncture,

- Mindfulness,

- Exercise.

#### 3.5.3 Protocols Focused on the Oncological Treatment of the Patient

- Radiofrequency ablation (RFA/Rhizotomy),

- Delayed chemotherapy,

- Surgery combined with chemo and radiotherapy.

#### 3.5.4 Special Treatment Protocols

- Parenteral nutrition,

- Paracentesis.

#### 3.5.5 More Advanced Protocols of Care

- Advanced care plans (multiple or combined palliative care protocols),

- Hospitalization or home hospitalization,

- Multidisciplinary palliative care.

Only a minority of these protocols were tested for effectiveness, either *via* comparisons between patients with and without palliative care or measuring differences between treatments administered independently or with the support of medical staff. Indeed, only 5 out of 40 articles reported no information on the control group [7, 30, 13, 38, 40], and only the last two studies reported numerical data concerning QoL.

These treatments produced improvements that vary between zero, minimal and discrete in several sub-categories that were cited in the results of the articles reviewed. Only 27 of the 40 articles reported numerical data regarding the results of the studies that were carried out; the remaining 13 studies reported a description of the results but no numerical data, and of these, only 4 reported the impossibility of arriving at a valid result due to “high attrition rate in the measurement of quality of life due to patient death” [22], “lack of focus and content analysis” [18], or because “the heterogeneity of QoL assessments makes direct comparisons difficult” [16].

When some improvement was reported, it concerned various dimensions of HR-QoL, in particular, physical, emotional, cognitive, mental, spiritual, social, vitality, general health, self-efficacy and optimism, personal autonomy, concerns about the quality of life, purpose in life, health care, and pain reduction (Table **1**).

## 4 DISCUSSION

The investigation of QoL is essential to understand the aspects (physical, mental, or functional) that are most affected in patients accessing palliative care and to better understand how to deal with them. This area of investigation is understudied despite being a relevant topic in evaluating the usefulness of palliative care in terminal patients. In many studies, the investigation of QoL represents a secondary or even marginal outcome. In most studies, the most relevant improvements concern the mental or functional aspects and, to a lesser extent, the physical ones.

The most noticeable improvements in HRQoL were especially in the studies comparing palliative care *versus* control groups without palliative care, with common medical treatments or without any treatment, supporting the theory on the efficacy of palliative care in relation to the conditions of terminal patients or patients with permanent disability [5, 6, 8, 11, 32, 33, 14, 34, 17, 42].

Some specific treatment was found to improve QoL in special groups of patients. For example, Rosian *et al*. reported in their study a noticeable improvement in bone pain in patients with metastatic cancer undergoing rhizotomy. An improvement in general QoL was reported by Burlacu *et al*. [44] in dying patients experiencing some sort of religious beliefs. They found fewer symptoms of depression and a lower risk of suicide in relation to a positive correlation with hope and spirituality, thus linking religiosity with a possible association with better mental health [44]. According to this study, there could be a strong correlation between religiosity and QoL improvement. Overall, improvement in QoL was more likely for medical or psychosocial protocols applied to patients with cancer, while other terminal conditions accessing palliative care were less likely to benefit from the administered protocols of care. This is an area in need of better trials, especially trials that test the proposed treatment against adequate control groups. There is a shortage of RCTs as far as QoL in palliative care is concerned.

It should be noted that the quality of the studies had a strong influence on the chance that some improvement in QoL was found in relation to palliative care. Based on the quality assessment and the results reported by the studies that received an excellent rating (25 studies with ++ scoring), there was a significant improvement in 20% of the reviewed studies, a non-significant improvement in 60%, and no improvement in 20%. Of the studies that received a positive but not excellent evaluation (25 studies), 40% of the studies reported a significant improvement, 33.3% reported a non-significant improvement, and 33.3% stated no improvement.

## CONCLUSION

Overall, the investigation of QoL in palliative care remains understudied. In many studies, QoL is a secondary outcome, and there is some tendency to use a disparate range of tools to measure it, whose reliability and validity should still be established in some groups of patients. There is some evidence that patients undergoing palliative care may benefit from it as far as QoL is concerned, especially in mental and functional areas.

## Figures and Tables

**Table 1:** General characteristics of the studies and main findings.

S.No.	**Study**	**Type of Study**	**Location**	**Sample**	**Condition**	**Diagnosis**	**Evaluation Tools**	**Main Results**
1	Salakari MR *et al*., 2015 [5]	Systematic Review	Europe North America Asia	K=13 N=1169	Patients with cancer *vs*. patients with cancer with common palliative therapy	Locally advanced incurable cancer	N/A	Exercise interventions positively impact various aspects of quality of life (QoL). Moreover, effective rehabilitation interventions have been linked to improvements in overall QoL. Overall, despite the limited evidence available, especially for patients with advanced cancer, current indications suggest that rehabilitation may be beneficial and can be recommended for patients with cancer as a chronic condition receiving palliative care.
2	Kavalieratos *et al*., 2016 [28]	Meta-analysis and Systematic Review	N/A	K= 86 N= 12731	Patients with life-limiting illness *vs*. patients with life-limiting illness without improved advance care planning	-Cancer - heart failure - Mixed - HIV - multiple sclerosis	- FACIT-Pal - Euro Qol EQ 5D; - FACT-G - Mqol- HK	Palliative care improved patient quality of life (QOL) and symptom burden at the 1- to 3-month follow-up (standardized mean difference, 0.46; 95% CI: 0.08 to 0.83 and −0.66; −1.25 to −0.07, respectively). The association between palliative care and QOL remained statistically significant in the 5 trials with a low risk of bias (0.20; 0.06 to 0.34) but not with symptom burden (−0.21; −0.42 to 0.00). No association with survival was found. However, palliative care consistently correlated with patient and caregiver satisfaction and reduced healthcare utilization. After correcting for the risk of bias, the impact of palliative care on QOL remained statistically significant, but its clinical significance was not substantial.
3	McCaffrey N *et al*., 2016 [6]	Systematic Review	- Australia - England - North America - Germany - New Zealand - Netherlands - South Africa - Sweden - Korea	K= 24 N= 467	Patients with advanced disease in PC *vs*. patients with advanced disease without PC	-Cancer - Heart failure - HIV - COPD - Mixed	-EORTC- QLQ- C30 - Euro Qol EQ 5D - FACIT – PAL - SF6- D - HUI 3 - PCOM - POS-E - ICECAP - SMC	The review traced several aspects of quality of life (QOL) important for individuals receiving palliative care, from physical and personal autonomy to emotional, cognitive, social, and spiritual well-being, until healthcare use and end-of-life preparatory.
4	Lau CH *et al*., 2016 [29]	Meta-analysis and Systematic Review	- China - Germany - Taiwan	K= 13 N= 1127	Patients with cancer *vs*. patients who have used any type of intervention without acupuncture	Non-small cell lung cancer -Gastrointestinal cancer - Various types of cancer	N/A	The results of the meta-analysis showed a modest improvement in quality of life among patients with gastrointestinal cancer after a combination of acupuncture and Chinese herbal medicine (n=111, standardized mean difference: 0.75, 95% CI: 0.36–1.13). A combination of acupoints injection of Astragalus extract and 3-step analgesic ladder medications showed superiority compared to only 3-step analgesic ladder medications in patients with non-small cell lung cancer (33.3% *versus* 14.3%, RR: 2.00, 95% CI: 1.46–8.70).
								Conversely, an isolated RCT (n=60) found no difference in quality of life among cancer patients treated with electroacupuncture, hydroelectric baths, vitamin B, or vitamin B placebo.
5	Maharaj S *et al*., 2016 [7]	Systematic Review	Caribbean	K=9 N= 312	Patients with advanced disease (control group non-specified)	-Advanced cancer -End-stage renal -Moribund intensive care	N/A	Just 2 out of 9 studies reported data on QoL. In a Jamaican cohort, higher quality of life scores were predicted by younger age, race, higher urea reduction ratio, and higher serum hemoglobin. Better quality of social interactions and energy scores were observed in people with the highest income. In another study, the inability to access affordable and nourishing foods limited the patient’s ability to enjoy a fair quality of life.
6	Health Quality Ontario, 2016 [8]	Systematic Review	West Europe North America Korea China Turkey	K=150 N=4,235	Patients with cancer *vs*. patients with cancer with usual care	- Cancers that metastasize to the spine - Primary cancers	- SF-36	Palliative care positively impacted the mean physical component summary score of the Short-Form Health Survey in patients with terminal multiple myeloma, producing an increase from 22.1 (range 20–25) at baseline to 41.8 (range 38–45) at the end of treatment, with results maintained at the 1-year follow-up.
7	Hayley Barnes *et al*., 2016 [9]	Systematic Review	N/A	K=26 N=526	Advanced disease and terminal illness *vs*. placebo	Refractory breathlessness	N/A	Four studies examined the effects on quality of life, but only one study provided usable data. A placebo-controlled trial of morphine in patients with chronic respiratory diseases found no difference between morphine and placebo (standardized mean difference: -0.86; 95% CI -9.9 to 8.18). However, there was some evidence that participants may have felt less in control when using morphine.
8	Waldemar Siemens *et al*., 2016 [10]	Systematic Review	N/A	K=50 N=1916	Patients with advanced disease *vs*. placebo	Pruritus	- Short Form 36 - VAS - SCIID - IDS-SR30	Four studies investigated quality of life as a secondary outcome, with only two controlled trials.
								In participants treated with colesevelam for chronic pancreatitis, one study showed no significant changes in various domains of quality of life (p>0.05). Another randomized controlled trial on 50 participants found that low-dose flumecinol produced a median improvement in quality of life compared to placebo, a finding supported by moderate-quality evidence. However, the higher dose of flumecinol did not lead to a significant improvement in quality of life.
9	Kun Hyung Kim *et al*., 2016 [11]	Systematic Review	China Taiwan Poland Iran	K=24 N=1787	Patients with advanced disease *vs*. routine care, conventional and sham intervention (some unspecified)	-Chronic kidney disease	-KDQOL - WHOQOL-BREF - SF 36	Four studies report QoL outcomes in palliative care. Better QoL compared to routine care was observed on the KDQOL sub-domains of physical functioning (1 study, 174 participants), vitality (2 studies, 174 participants), and general health (2 studies, 174 participants). The outcome was measured three to four months from baseline. In a six-month follow-up observation of moxibustion treatment, favorable effects compared with routine care were found on the following sub-domains: physical functioning (109 participants), vitality (109 participants), and cognitive function (109 participants).
10	Guerrero-Torrelles M *et al*., 2017 [30]	Systematic Review and Realist Synthesis	- North America - Israel - Japan - Australia - China	K= 12 N= 359	Patients with advanced cancer (control group non-specified)	-Lymphoma - Ovarian Cancer - Solid and Hematological Tumor	- QOLC - MQOL - QOL- E - FACTG	The review examined 12 studies, with 4 of them exploring quality of life in palliative care. Several improvements were noted in specific domains of quality of life, such as purpose in life and spiritual well-being, self-efficacy and optimism, and concerns related to quality of life.
11	Gaertner J *et al*., 2017 [31]	Meta-analysis and Systematic Review	N/A	K= 12 N= 2454	Adults with advanced incurable illness *vs*. adults with advanced incurable illness in multidisciplinary support and telephone palliative care	-Advanced cancer - Post. Op. - ICU Patients - Acute heart failure	-MQOLS-CA - EORTC QLQ-C30 - FACTG	In six trials (n=1218), specialist palliative care shows a modest beneficial effect (standardized mean difference: 0.16; 95% CI: 0.01 to 0.31; I2=38%, moderate quality evidence), with an effect of 4.1 (0.3 to 8.2) on the EORTC QLQ-C30 global health/QoL scale. The effect favoring specialist palliative care was slightly higher for cancer patients (0.20; 0.01 to 0.38; n=828, five trials) and highest for early care (0.33; 0.05 to 0.61; n=388, two trials). The effects on the EORTC QLQ-C30 global health/QoL scale were 5.1 (0.3 to 9.7) and 8.5 (1.3 to 15.6), respectively.
12	Mochamat *et al*., 2017 [12]	Systematic Review	N/A	K= 21 N= 1940	Cancer patients suffering from cachexia or cachexia-related symptoms *vs*. placebo and no supplementation of vitamin C	- Testicular; Ovarian Cancer - Primary Neoplasm - Prostate cancer - Pancreatic cancer - Intra- abdominal carcinoma - Head and neck- cancer - Gastrointestinal cancer - Colon, rectum cancer - Squamous cell carcinoma of the oral cavity, pharynx, larynx - Gastric cancer	- EORTC - Euro QoL EQ-5D	Out of the 21 studies included in this review, four specifically examined the impact of vitamins, minerals, and proteins on the quality of life in palliative care. The findings revealed that oral and intravenous supplementation of vitamin C among terminal cancer patients resulted in enhancements across various aspects of their quality of life.
13	Schuurhuizen CSEW *et al*., 2017 [13]	Systematic Review	N/A	K= 30 N= 19863	Patients with metastatic colorectal cancer (control group non-specified)	N/A	- EORTC- QLQ-C30 - Euro Qol EQ 5D - FACT-C	In 25 out of the 30 trials analyzed (83%), there was no discernible difference in global quality of life between the treatment arms.
14	Diop MS *et al*., 2017 [32]	Meta-analysis and Systematic Review	- America	K= 15 N= 24.403	Patients with heart-failure *vs*. patients with heart-failure with no PC interventions	N/A	- CHQ - MLHF-Q	Out of the 15 studies examined in this review, six focused on quality of life in palliative care. Patient quality of life significantly improved in 83% (5 out of 6) of these studies.
15	Wang CW *et al*., 2017 [33]	Meta-analysis and Systematic Review	- Hong Kong - Japan - Portugal - United Kingdom - United States	K = 8 N = 955	Terminal or advanced cancer *vs*. no psychological intervention or placebo intervention	N/A	- FACIT - SP- M - HADS (Hospital - Anxiety and depression scale) - MQOL - QOLC- E	The impact of life review therapies on quality of life (QOL) was investigated in six randomized controlled trials (RCTs). QOL was assessed with validated scales in five RCTs, while four RCTs used single-item or two-item scales.
								Pooled results showed a statistically significant effect size for overall QOL measured with single-item or two-item scales at both post-intervention (standardized mean difference = 0.35; 95% CI: 0.15 to 0.56; p < 0.001) and follow-ups (0.82; 0.47 to 1.18; p < 0.0001), albeit with high heterogeneity (I2 = 91% and 90%, respectively). After excluding one trial (Xiao *et al*.), the pooled effect sizes for overall QOL became insignificant at post-intervention (0.10; −0.13 to 0.32; p = 0.40; I2 = 0%) and follow-ups (0.04; −0.45 to 0.53; p = 0.88; I2 = 0%). The pooled effect size on the total scores of the validated QOL scales was not statistically significant (0.25; −0.03 to 0.54; p = 0.08).
16	Vincent T Janmaat *et al*., 2017 [14]	Systematic Review	Asia, Europe, North America, South America, Australia, and the ex-Soviet Union block of nations	K=11 N=1347	Patients with cancer *vs* patients with cancer with common or conventional treatment or placebo	Esophageal and gastroesophageal junction cancer	N/A	Only five studies addressed quality of life (QoL), but they were not representative of all the studies analyzed; four of them tested a targeted agent, and four did not report data separately for the esophageal and GE-junction cancer subgroups. Overall, QoL showed improvement in the arms with the add-on agent. One study indicated a not statistically significant trend toward better quality of life (measured by QLQ-C30) at six weeks for participants in the ramucirumab group compared to those in the placebo group (p = 0.23).
17	Kassianos AP *et al*., 2017 [34]	Meta-analysis and Systematic Review	North America, Norway, Netherlands, Japan, Turkey	K=11 N=2939	Patients with cancer *vs*. patients with cancer with usual care	Primary and metastatic cancer	-SEIQoL-DW -EORTC QLQ C-30 -FACIT-pal -FACT-L -FACIT-sp -QUAL-E -McGill QoL Questionnaire	Specialist palliative care demonstrated a moderately positive impact on health-related quality of life (HRQoL) (standardized mean difference: 0.28; 95% CI: 0.16 to 0.41; p < 0.001), with a marginally significant publication bias favoring studies with positive effect sizes (Kendall’s tau = 0.673, p = 0.004).
								Differences were non-significant between RCTs and non-RCTs (p = 0.990), cancer types (p = 0.627), and among inpatients, outpatients, and both (p = 0.172). However, mixed-effects analysis revealed a positive impact of specialist palliative care in studies involving inpatients (0.55; 0.17 to 0.92; p = 0.004) or both (0.18; 0.08 to 0.27; p < 0.001) but not for outpatients (0.20; −0.03 to 0.44; p = 0.89). Meta-regression analyses indicated that patients’ age and treatment duration were insignificant predictors of the overall effect size on HRQoL. Heterogeneity was partially accounted for by differences across the specialists delivering the intervention.
18	Latorraca COC *et al*., 2017 [25]	Systematic Review	USA Malaysia Hong Kong	K= 4 N=234	Adults (over 18 years old) in palliative care *vs* patients not performing mindfulness	-(COPD) Chronic obstructive pulmonary disease -Cancer	-SF-36 -SF-36 for Veterans (VR-36)	In a study comparing mindfulness intervention over eight weeks, with one session per week plus daily individual practice, *versus* a control group (support group) for patients with chronic obstructive pulmonary disease, the control group showed a statistically significant advantage in quality of life regarding physical aspects measured by Short Form-36 for Veterans (VR-36) (MD: −4.30; 95%CI: −7.99 to −0.6; participants: 49; low-quality evidence). However, no other outcomes analyzed by this study exhibited statistically significant differences.
								These outcomes included quality of life sub-scores for activity (SGRQ), symptoms (SGRQ), impact (SGRQ), and mental aspects.
19	Dittus KL *et al*., 2017 [16]	Systematic Review	North America Europe Taiwan Australia	K=26 N=2153	Patients with cancer *vs*. patients who do not receive exercise intervention	Advanced metastatic cancer	N/A	The extensive heterogeneity in the quality of life (QOL) assessments makes impossible comparisons across reviewed studies.
20	Grossman CH *et al*., 2018 [17]	Systematic Review	- North America - China - Australia - Japan	K= 9 N= 1179	Patients with advanced cancer *vs*. patients with usual care, no intervention or other control.	- Solid organ tumors - Ovarian cancer -Incurable cancer	- MQOL - DAS - DADDS - QOLC-E - FACIT SP	Out of the 9 studies analyzed, 4 focused on assessing the quality of life (QOL) in palliative care. Among them, one study revealed significant enhancements in QOL (p < 0.01), while another indicated a trend toward improvement that did not reach statistical significance (p = 0.07). Additionally, a third study reported substantial improvements in QOL (p < 0.05), while the fourth highlighted a notable reduction in 'distress about dying' among terminal patients (p = 0.04).
21	Van Roij J *et al*., 2018 [18]	Systematic Review	N/A	K= 69 N= 3282 participants (21,077 participants in total)	Life-threatening illnesses *vs*. patients with threatening illness *vs*. non-self-administered instruments patients	-Heart failure -End-stage lung disease -Advanced renal disease -Late-stage Parkinson's disease -Cancer	- EORTC QLQ (1-40 items) - ESAS - MQOL	The evaluation of Health-Related Quality of Life (HRQOL) lacks focus and comprehensive content analysis. None of the measurement instruments underwent adequate assessment for all measurement properties, and the studies failed to adequately evaluate certain crucial psychometric properties. Many measurement instruments exhibited shortcomings, such as insufficient recall time or a lack of measurement across all HRQOL areas.
22	Schüchen RH *et al*., 2018 [35]	Meta-analysis and Systematic Review	N/A	K= 43 N= 2925	Patients with end-stage medical disease *vs*. patients with end-stage medical disease without opioids	Cancer pain	N/A	The focus of the reviewed studies centered on managing cancer pain. Two studies found no significant difference in the quality of life index when comparing the combination of acetaminophen and strong opioids to placebo. However, one study reported a slight advantage in overall well-being (p = 0.05). Similarly, there were no significant differences observed between dexketoprofen, trometamol, or ketorolac.
								Another study indicated that analgesia with flurbiprofen, combined with other opioids, resulted in a significantly higher Karnofsky score (p = 0.05). However, this finding's significance is limited due to a very small sample size.
23	Rosian K *et al*., 2018 [19]	Systematic Review	Germany USA Japan	K=9 N=640	Patients with advanced disease *vs*. patients who got pain reduction after being treated with RFA	Bone metastasis	- FACT-G7 - FACT-BP	Out of the nine studies examined, only two addressed Quality of Life (QoL). These two studies indicated that Radiofrequency Ablation (RFA) results in significant pain reduction and improvement in Health-Related Quality of Life (HRQoL). They reported statistically significant improvements over one month and three months of treatment, with FACT-G7 scores increasing by 4.8 and 5.2, respectively, and FACT-BP scores increasing by 14.7 and 16.3, respectively, compared to baseline.
24	Claassen YH *et al*., 2018 [20]	Systematic Review	N/A	K= N= 176	Patients with cancer with immediate chemotherapy *versus* delayed chemotherapy	Metastatic colorectal cancer	-EORTC QLQ	Two studies provided data on quality of life but did not reveal a clear distinction between the study arms. Moreover, the number of participants was not provided. The relevance of the findings was dubious.
25	Omar Abdel‐Rahman *et al*., 2018 [21]	Systematic Review	N/A	K=2 N=104	One trial compared the addition of surgery and radiotherapy to chemotherapy with chemotherapy alone. The other trial compared the addition of radiotherapy to chemotherapy and surgery with chemotherapy and surgery alone	Malignant pleural mesothelioma	- EORTC QLQ-C30 - EORTC QLQ-LC13	Two trials were examined. There were no changes in the scores for the overall evaluation of life in either group up to week 14 after randomization, and no statistically significant differences were observed between the treatment groups.
26	Fulton JJ *et al*., 2018 [43]	Meta-Analysis	N/A	K= 32 N=1536	Depression and anxiety in terminal illness *vs* patients under psychotherapy	-Cancer -Multiple sclerosis -HIV/AIDS -Advanced and terminal illness	-MQoL -QoL Scale -Functional LivingIndex -EQ-5D -FACT-G -EORTC-QoL -QUAL-E	Overall, psychotherapy led to a notable increase in Quality of Life (QoL), albeit with a small effect size of 0.47 (95% CI: 0.17 to -0.78; I2=89%). However, the findings suggest a potential publication bias in studies exploring depression and QoL.
								Additionally, the majority of identified studies have concentrated on cancer patients, and the results cannot be generalized to interventions for other conditions.
27	Sowerbutts AM *et al*., 2018 [22]	Systematic Review	North America Italy Israel England	K=13 N=721	Patients with cancer *vs*. QoL derived from PN	-Malignant bowel obstruction	-EORTC QLQ-C30	QoL data were only reported in four studies. However, there was a high attrition rate in the measurement of quality of life, primarily due to patient mortality. Quality of life was assessed at four months in less than half of the participants. The relevance of the findings was dubious.
28	Gao Y *et al*., 2019 [36]	Meta-analysis and Systematic Review	- Germany - China - America - Australia	K= 11 N= 969	Terminally Ill patients *vs* QoL of patients under music therapy	- Advanced cancer - Congestive heart failure - Chronic renal failure	- EORTC	The meta-analysis revealed a significant improvement in Quality of Life (QoL) among participants receiving music therapy (standardized mean difference: 0.61; 95% CI: 0.41 to 0.82, p < 0.00001; heterogeneity: I2 = 73%, p < 0.05) compared to those receiving general palliative care. Subgroup analysis using the EORTC scale demonstrated that music therapy could enhance the QoL of terminally ill patients (0.29; 0.03 to 0.55, p = 0.03; heterogeneity: I2 = 0%, p = 0.46), with even more remarkable results in the HRQOL subgroup analysis (1.07; 0.76 to 1.38; p < 0.00001; heterogeneity: I2 = 0%, p = 0.78).
29	Fulton JJ *et al*., 2019 [37]	Meta-analysis and Systematic Review	- North America - Denmark - Europe	K= 10 N= 2385	Patients with advanced cancer *vs*. effectivity of palliative care	- Lung cancer - Gastrointestinal cancer - Breast cancer - Genitourinary cancer - Head/neck cancer - Pancreatic cancer	- FACT-G - FACIT- SP - FACT-PC - FACT-TOI	Integrated palliative care demonstrated a significant improvement in short-term quality of life (k=9; standardized mean difference: 0.24; 95%CI: 0.13 to 0.35; I2 = 0.0%). The positive effects were consistent across studies, ranging from small to moderate in all but one. However, at 6–12 months, there was no observed improvement in quality of life (k = 6; 0.15; –0.12 to 0.43; I2 = 28%).
30	Cui X *et al*., 2019 [38]	Meta-analysis and Systematic Review	- North America - Sweden - China - United Kingdom - Northern Ireland	K= 21 N= 2999	Patients with chronic heart failure (control group not specified)	N/A	N/A	The chronically critically ill group exhibited significantly improved QoL compared to the routine care group (standardized mean difference=0.60; 95%CI: 0.27–0.94; I2=94%). Further subgroup analyses were conducted to evaluate the sources of heterogeneity. Similarly, patients receiving multidisciplinary intervention (0.63; 0.14–1.11; I2=92%) and those undergoing non-multidisciplinary intervention (0.59; 0.11–1.06; I2=95%) also showed significantly improved QoL; however, significant heterogeneity was observed in the comparison groups. Notably, patients receiving face-to-face interventions experienced a significant improvement (0.54; 0.24–0.85; I2=89%) in QoL compared to those who received telephone-only interventions.
31	Friedel M *et al*., 2019 [23]	Systematic Review	Europe North America Lebanon	K= 19 N= 1082	Life-limiting diseases﻿*vs* PPC interventions ranged from home-based to hospital and respite care	N/A	- PedsQL 4.0 - QOLLTI-F - HADS - Needs at the End of Life Screening Tool	Among the studies, a total of 23 different instruments were identified, including the PedsQL 4.0 used in 3 studies, QOLLTI-F in 2 studies, SCCC in 2 studies, and HADS in 2 studies. All of these instruments were standardized measures. The Standard Error of Measurement (SEM) varied, ranging from 0.38 (with 95% CI = ± 0.74) for the QOLLTI-F, which has a scale from 0 to 70, to 6.27 (with 95% CI = ± 12.29) for the PedsQL 4.0, which has a scale from 0 to 100.
32	Ibeneme SC *et al*., 2019 [39]	Meta-Analysis and Systematic Review	USA Brazil Rwanda Nigeria South Africa	K=19 N= 491	Differences between patients who do aerobic exercise and those who do not	HIV/AIDS(PLWHA).	- MOS-HIV survey CD4 Count - SWB -EQ5D -SF-36 -WHOQOL-BREF	In the reviewed studies, Quality of Life (QoL) was investigated in a series of Randomized Controlled Trials (RCTs). Among them, five studies utilized aerobic exercise as the intervention, three studies utilized resistance exercise, and two studies combined both interventions. In the aerobic exercise and combined studies, the control groups were subjected to no exercise, maintenance of daily activity, and short-wave diathermy as a placebo, with counseling groups serving as controls. Conversely, in the resistance exercise studies, control groups were subjected to no exercise, usual advice, and normal activities. Additionally, one of the three resistance exercise studies assessed the effects of co-intervention with progressive resistance exercises and whey protein, with a comparison group receiving whey protein only. A non-statistically significant standardized mean difference was found (1.57; 95%CI: -4.97 to 1.83; I^2^= 97%).
33	Chumnan Kietpeerakool *et al*., 2019 [25]	Systematic Review	Europe	K=1 N=245	Woman patients under drainage treatment combined with catumaxomab *versus* drainage alone	Malignant ascites in gynecological cancer	- EORTC QLQ-C30	The findings were inconclusive in evaluating the disparity between the reviewed treatments. While women receiving drainage combined with catumaxomab demonstrated prolonged improvement in quality of life compared to those receiving drainage alone, the evidence is uncertain due to the limited number of participants and trials. The global quality of life showed a standardized mean difference of 0.17 (95% CI: 0.10 to 0.28).
34	Carolina OC Latorraca *et al*., 2019 [15]	Systematic Review	Italy UK	K=3 N=146	Patients with advanced disease with multidisciplinary, fast-track palliative care versus multidisciplinary standard care while on a waiting-list control	Multiple sclerosis	- SEiQOL-DW	A single study on Health-related quality of life (HR-QOL) with 64 participants provided some evidence of very low certainty. SEIQoL scores, where higher values denote better quality of life, demonstrated a not statistically significant mean difference at the end of treatment of 4.80 (95% CI: -12.32 to 21.92).
35	Evan T. Hall *et al*., 2019 [26]	Systematic Review	N/A	K=15	Patients with cancer *vs*. cancer patients receiving ICIs as compared to other anticancer therapies.	-Melanoma -Lung cancer -Genitourinary cancer -Head/neck cancer	-EORTCQLQ-C30 -EORTCQLQ-LC13 -EORTC QLQ-H&N35 -LCSS -EQ-5D-3L -FKSI-19 -FKSI-DRS -EQ-5D	In this review of Patient-Reported Outcomes (PROs) in Immune Checkpoint Inhibitor (ICI) trials involving cancer patients, overall Health-Related Quality of Life (HRQoL) varied from being similar to slightly improved among those treated with ICIs compared to other cancer therapies. Symptom scales commonly assessed, such as fatigue, gastrointestinal symptoms, and pain, showed comparable outcomes between ICIs and alternative cancer treatments despite significant rates of high-grade Immune-Related Adverse Events (IRAEs) reported by clinicians during these trials.
36	Zhou K *et al*., 2019 [42]	Meta-analysis	N/A	K=7 N=769	Heart failure *vs*. usual care for heart failure compared to palliative care	Advanced and chronic heart failure	N/A	In comparison to usual care for heart failure patients, palliative care demonstrated a significant improvement in quality of life (standardized mean difference = 1.46; 95% CI: 0.12 to 2.79; p = 0.03; I2=96%), yet it did not impact rehospitalization rates (relative risk = 0.84; 0.66 to 1.07; p = 0.16). Additionally, palliative care showed a notable reduction in depression scores among heart failure patients (–0.62; –0.99 to –0.25; p = 0.03).
37	Tobberup R *et al*., 2019 [27]	Systematic literature review	Denmark, Korea, Italy, USA, Spain, Germany	K=8 N=233	Patients with cancer *vs*. patients under anti-neoplastic treatment in who PN treatment is the only feeding opportunity, but not necessarily in patients able to feed enterally.	-Gastric cancer -Colorectal cancer -Pancreatic cancer -Gynecological cancers	-EORTC QLQ-C15 PAL -EORTC QLQ C-30	In one Randomized Controlled Trial (RCT), a significantly higher mean score of +16 points (95% CI: 0.6 to 31) in Health-Related Quality of Life (HRQoL) at 12 weeks favored Parenteral Nutrition (PN) over control treatment (p < 0.05), although no significant differences were observed at weeks 6, 18, or 24. In an observational study, HRQoL remained unchanged after one month but showed significant improvement after two months (+12 points, p=0.02) and three months (+24 points, p=0.02). Another observational study reported significant improvement over four months using repeated measures analysis (p < 0.001), with increases of +6 points at one month, +14 points at two months, +19 points at three months, and +14 points at four months. In summary, the impact of current PN treatment on HRQoL in patients with advanced cancer remains inadequately investigated.
38	Warth M *et al*., 2019 [41]	Meta-analysis and Systematic Review	North America Asia Europe Australia	K=15 N= 1248	Patients with cancer *vs*. improvement of QoL for patients who use life review techniques and music therapy	-Primary cancer diagnosis -Advanced terminal cancer	-Single-item scale -MQOL -two-item scale -HQLI-R (overall) -distress thermometer	In the reviewed studies, a statistically significant medium-sized overall effect of d = 0.73 (95% CI: 0.15 to 1.30, p = 0.02) favored psychosocial interventions in the random-effects model but with high heterogeneity (I2=91%). No potential moderator was found to significantly explain variance across studies. Further examination of model diagnostics identified two highly influential studies (d = 1.82 and d = 2.61) with very large effect sizes. Even after excluding these outliers, an improvement in quality of life was found, with a standardized mean difference of -0.36 (95% CI: -0.08 to -0.64) with moderate heterogeneity (I2 = 73%).
39	Burlacu A *et al*., 2019 [45]	Systematic Review	America, Europe, Philippines, Thailand, Malaysia, Taiwan	K=50 N= 9265	Dialysis patients *vs*. use of R/S assessment in dialysis patients	-Hemodialysis and peritoneal dialysis	-WHOQOL-brief -WHOQOL-SRPB -HRQoL-Ferrans and Powers QLI Dialysis Version-III - KDQOL-SF -WHOQOL -SF-36 -SWLS -EQ-5D-3L	In this review, nineteen studies (comprising 9 RCTs and 10 single-arm studies) explored the impact of exercise on enhancing Quality of Life (QOL) among individuals with advanced cancer. Among these studies, 10 (52.6%) reported an improvement in QOL, while 9 (47.4%) found no significant change; notably, the positive studies had larger participant numbers. Additionally, this review underscores the strong correlation between religiosity and enhanced QOL. Plausible explanations for this notable finding include reduced symptoms of depression, a decreased risk of suicide, positive associations with hope and spirituality, and potential links to improved mental health.
40	Lu F *et al*., 2019 [40]	Meta-analysis and Systematic Review	USA India Mexico Canada	K=6 N=437(60 for the study that reported QoL data)	Patients with cancer (control group not specified)	-Pancreatic cancer -Abdominal cancer -Abdominal pain -Celiac plexus neurolysis	N/A	Only one study provided Quality of Life (QoL) data, revealing no significant difference between the two investigated groups at 3 months.

**Table 2:** Quality evaluation of the studies. The evaluation was done according to the assessment of multiple systematic reviews (AMSTAR) scale.

S.NO	Study	1.1	1.2	1.3	1.4	1.5	1.6	1.7	1.8	1.9	1.10	1.11	2.1
1	1.Salakari MR et al., 2015 [5]	Yes	Yes	Yes	Yes	Yes	No	Yes	Yes	No	No	Yes	+
2	2.Kavalieratos et al., 2016 [28]	Yes	Yes	Yes	Yes	Yes	Yes	Yes	Yes	Yes	Yes	Yes	++
3	3.McCaffrey N et al., 2016 [6]	Yes	Yes	Yes	Yes	Yes	Yes	No	No	Cs	No	Yes	+
4	4. Lau CH et al., 2016 [29]	Yes	Yes	Yes	Yes	Yes	Yes	Yes	Yes	Yes	Yes	Yes	++
5	5. Maharaj S et al., 2016 [7]	Yes	Yes	Yes	Yes	Yes	Yes	Yes	Yes	Cs	Cs	No	+
6	6. Health Quality Ontario. 2016 [8]	Yes	Yes	Yes	Yes	Yes	Yes	Yes	Yes	No	Yes	No	+
7	7. Hayley Barnes et al., 2016 [9]	Yes	Yes	Yes	Yes	Yes	Yes	Yes	Yes	Cs	Yes	Yes	++
8	8. Waldemar Siemens et al., 2016 [10]	Yes	Yes	Yes	Yes	Yes	Yes	Yes	Yes	Cs	Cs	Yes	++
9	9. Kun Hyung Kim et al., 2016 [11]	Yes	Yes	Yes	Yes	Yes	Yes	Yes	Yes	Yes	Yes	Yes	++
10	10. Guerrero-Torrelles M et al., 2017 [30]	Yes	Yes	Yes	Yes	Yes	Yes	Yes	No	Yes	No	Yes	+
11	11. Gaertner J et al., 2017 [31]	Yes	Yes	Yes	Yes	Yes	Yes	Yes	Yes	Yes	No	Yes	++
12	12. Mochamat et al., 2017 [12]	Yes	Yes	Yes	Yes	Yes	Yes	Yes	Yes	Cs	No	Yes	++
13	13. Schuurhuizen CSEW et al., 2017 [13]	Yes	Yes	Yes	Yes	Yes	Yes	Yes	Yes	Cs	No	Yes	+
14	14. Diop MS et al., 2017 [32]	Yes	Yes	Yes	Yes	Yes	Yes	No	No	Yes	No	Yes	+
15	15. Wang CW et al., 2017 [33]	Yes	Yes	Yes	Yes	Yes	Yes	Yes	Yes	Yes	No	Yes	++
16	16. Vincent T Janmaat et al., 2017 [14]	Yes	Yes	Yes	Yes	Yes	Yes	Yes	Yes	Cs	Yes	Yes	++
17	17. Kassianos AP et al., 2017 [34]	Yes	Yes	Yes	Yes	Yes	Yes	Yes	Yes	Yes	Yes	Yes	++
18	18. Latorraca COC et al., 2017 [25]	Yes	Yes	Yes	Yes	Yes	Yes	Yes	Yes	Cs	No	Yes	+
19	19. Dittus KL et al., 2017 [16]	Yes	Yes	Yes	Yes	Yes	Yes	No	No	Yes	No	Yes	+
20	20. Grossman CH et al., 2018 [17]	Yes	Yes	Yes	Yes	Yes	Yes	Yes	Yes	Cs	No	Yes	+
21	21. Van Roij J et al., 2018 [18]	Yes	Yes	Yes	Yes	Yes	Yes	Yes	Yes	Cs	No	No	+
22	22. Schüchen RH et al., 2018 [35]	Yes	Yes	Yes	Yes	Yes	Yes	Yes	Yes	Yes	No	Yes	++
23	23. Rosian K et al., 2018 [19]	Yes	Yes	Yes	Yes	Yes	Yes	Yes	Yes	Yes	Cs	Yes	++
24	24. Claassen YH et al., 2018 [20]	Yes	Yes	Yes	Yes	Yes	Yes	Yes	Yes	Yes	Cs	Yes	++
25	25. Omar Abdel‐Rahman et al., 2018 [21]	Yes	Yes	Yes	Yes	Yes	Yes	Yes	Yes	Cs	Yes	Yes	++
26	26. Fulton JJ et al., 2018 [43]	Yes	Yes	Yes	Yes	Yes	Yes	Yes	No	Yes	Yes	Yes	++
27	27. Sowerbutts AM et al., 2018 [22]	Yes	Yes	Yes	Yes	Yes	Yes	Yes	Yes	Yes	Yes	Yes	++
28	28. Gao Y et al., 2019 [36]	Yes	Yes	Yes	Yes	Yes	Yes	Yes	Yes	Yes	No	Yes	++
29	29. Fulton JJ et al., 2019 [37]	Yes	Yes	Yes	Yes	Yes	Yes	Yes	Yes	Yes	No	Yes	++
30	30. Cui X et al., 2019 [38]	Yes	Yes	Yes	Yes	Yes	Yes	Yes	Yes	Yes	Yes	Yes	++
31	31. Friedel M et al., 2019 [23]	Yes	Yes	Yes	Yes	Yes	Yes	Yes	Yes	Cs	Cs	Yes	+
32	32. Ibeneme SC et al., 2019 [39]	Yes	Yes	Yes	Yes	Yes	Yes	Yes	Yes	Yes	No	Yes	++
33	33. Chumnan Kietpeerakool et al., 2019 [25]	Yes	Yes	Yes	Yes	Yes	Yes	Yes	Yes	Cs	Yes	Yes	++
34	34. Carolina OC Latorraca et al., 2019 [15]	Yes	Yes	Yes	Yes	Yes	Yes	Yes	Yes	Cs	Cs	Yes	++
35	35. Evan T. Hall et al., 2019 [26]	Yes	Yes	Yes	Yes	Yes	Yes	Yes	No	Yes	No	No	+
36	36. Zhou K et al., 2019 [42]	Yes	Yes	Yes	Yes	Yes	Yes	No	No	Yes	Cs	Yes	+
37	37. Tobberup R et al., 2019 [27]	Yes	Yes	Yes	Yes	Yes	No	Yes	No	Cs	Cs	Yes	+
38	38. Warth M et al., 2019 [41]	Yes	Yes	Yes	Yes	Yes	Yes	Yes	Yes	Yes	Yes	Yes	++
39	39. Burlacu A et al., 2019 [45 ]	Yes	Yes	Yes	Yes	Yes	Yes	Yes	Yes	Yes	No	Yes	++
40	40. Lu F et al., 2019 [40]	Yes	Yes	Yes	Yes	Yes	Yes	Yes	Yes	Yes	Cs	Yes	++

Note: 1.1: The study addresses a clearly defined research question.

1.2: At least two people should select studies and extract data.

1.3: A comprehensive literature search is carried out.

1.4: The authors clearly state if or how they limited their review by publication type.

1.5: The included and excluded studies are listed.

1.6: The characteristics of the included studies are provided.

1.7: The scientific quality of the included studies is assessed and documented.

1.8: The scientific quality of the included studies is assessed appropriately.

1.9: Appropriate methods are used to combine the individual study findings.

1.10: The likelihood of publication bias is assessed.

1.11: Conflicts of interest are declared.

2.1: What is your overall assessment of the methodological quality of this review? -High quality (++) -Acceptable (+) -Unacceptable – reject 0

**Table 3:** Tools that are most often used to evaluate the QoL in the reviewed studies.

Acronym	Complete Name	Description	Reference
EORTC QLQ (1-40 items)	European Organization for Research and Treatment of Cancer Quality of Life questionnaire	This is a 40-item questionnaire designed to evaluate the quality of life among cancer patients. It was translated into over 100 languages and is widely used.	• Van Roij J *et al*., 2018 [18] • Claassen YH *et al*., 2018 [20] • Abdel‐Rahman *et al*., 2018 [21] • Chumnan Kietpeerakool *et al*., 2019 [25] • Evan T. Hall *et al*., 2019 [26]
ESAS	Edmonton Symptom Assessment Scale	The ESAS targets nine prevalent symptoms in cancer patients, including fatigue, pain, nausea, depression, and anxiety. Symptom severity is graded from 0 to 10, with 10 indicating the utmost severity. The ESAS offers a framework for comprehending the onset and progression of symptoms.	• Van Roij J *et al*., 2018 [18]
EORTC QLQ-LC13	European Organization for Research and Treatment of Cancer Quality of Life Questionnaire Lung Cancer Module (EORTC QLQ-LC13)	This 13-item tool is intended specifically for lung cancer, to be used alongside the QLQ-C30 (see the EORTC QLQ-C30).	• Abdel‐Rahman *et al*., 2018 [21] • Evan T. Hall *et al*., 2019 [26]
EUROQOL EQ-5D	EuroQol Five-Dimensions Questionnaire	The EQ-5D encompasses five dimensions, each explored with a single question: mobility, self-care, usual activities, pain/discomfort, and anxiety/depression. It is a generic quality-of-life assessment tool developed in Europe but extensively applied worldwide.	• Ibeneme SC *et al*., 2019 [39] • Evan T. Hall *et al*., 2019 [26] • Fulton JJ *et al*., 2018 [43] • Burlacu A *et al*., 2019 [45]
FACT-BP	Functional Assessment of Cancer Therapy - Bone Pain	The FACT-BP is a 16-item questionnaire that includes a broader core module, FACT-General (FACT-G), and explores three key areas of quality of life in patients with bone metastases: general functioning, physical well-being, and bone pain.	• Rosian K *et al*., 2018 [19] • Evan T. Hall *et al*., 2019 [26]
FACT – G	Functional Assessment of Cancer Therapy - General	The FACT-G gauges the effects of cancer treatment across four domains: physical, social/family, emotional, and functional. There are supplementary questions to capture cancer-specific factors potentially influencing quality of life.	• Gaertner J *et al*., 2017 [31]
FACT-G7	Functional Assessment of Cancer Therapy - General (7-item version)	A short version of the FACT-G features three items from the physical well-being subscale (fatigue, pain, and nausea), one item from the emotional well-being subscale (concern about condition deterioration), and three items from the functional well-being subscale (life enjoyment, satisfaction with quality of life, and sleep).	• Rosian K *et al*., 2018 [19] • Kassianos AP *et al*., 2017 [34] • Fulton JJ *et al*., 2018 [43]
HADS	Hospital anxiety and depression	The HADS is a widely used 14-item tool to assess levels of anxiety and depression in patients. Seven items are on anxiety, and seven on depression.	• Friedel M *et al*., 2019 [23]
IDS-SR30	Self-rated Inventory of Depressive Symptomatology	The IDS-SR30, widely used in large-scale studies and clinical trials, assesses depression severity over seven days. Its clinician-rated (IDS-C) and self-report (IDS-SR) versions are easy to use and sensitive to treatment effects, making them valuable for research and clinical use.	• Waldemar Siemens *et al*., 2016 [10]
KDQOL KDQOL SF	Kidney Disease Quality of Life Instrument	The KDQOL survey expands the MOS SF-36 by focusing on kidney disease patients' HRQOL, incorporating specific items like symptoms, burden of illness, social interaction, staff support, and patient satisfaction.	• Kun Hyung Kim *et al*., 2016 [11] • Burlacu A *et al*., 2019 [45]
MQOL	The McGill Quality of Life Questionnaire	The tool is crafted to measure key areas of quality of life (physical, psychological, social, and occasionally existential/spiritual) pertinent to individuals facing life-threatening illnesses.	• Van Roij J *et al*., 2018 [18] • Kassianos AP *et al*., 2017 [34] • Fulton JJ *et al*., 2018 [43] • Warth M *et al*., 2019 [41]
MOS-HIV survey CD4 Count	HIV Medical Outcomes Survey	The HIV Medical Outcomes Survey assesses HRQOL among those with HIV. Its 35 items cover ten health dimensions and typically require about five minutes to complete.	• Ibeneme SC *et al*., 2019 [39]
NEST	Needs at the End of Life Screening Tool	The NEST includes 13 questions and screens for palliative care needs across four domains: social, existential, symptoms (physical and psychological), and therapeutic. An advantage of the NEST is that it has been validated in palliative care.	• Friedel M *et al*., 2019 [23]
PedsQL 4.0	Pediatric Quality of Life Inventory Version 4.0	This is a 23-item tool assessing HRQOL in children and adolescents, whether healthy or affected by acute and chronic health conditions. It has some generic core scales, investigating physical, emotional, social, and school functioning alongside disease-specific modules that are unified into a meaningful measurement system.	• Friedel M *et al*., 2019 [23]
QOLLTI-F	Quality of Life in Life-Threatening Illness – Family Caregiver Questionnaire	The QOLLTI-F is a 17-item multidimensional tool tailored to assess various aspects of family caregivers' experiences, including their state, distress related to the patient's condition, environment, outlook, financial concerns, relationships, quality of care, and overall quality of life. It has been developed from a qualitative study involving palliative care caregivers.	• Friedel M *et al*., 2019 [23]
SWB	Subjective wellbeing (SWB) measurement	The SWB is a self-reported 24-item measure of well-being. Its questions encompass emotional reactions, including infrequent negative affect, cognitive judgments, and life satisfaction.	• Ibeneme SC *et al*., 2019 [39]
SCID	Structured Clinical Interview for DSM Disorders	It is a 119-item semi-structured interview guide used to diagnose mental disorders based on criteria outlined in the American Psychiatric Association’s Diagnostic and Statistical Manual for Mental Disorders (DSM).	• Waldemar Siemens *et al*., 2016 [10]
SEiQOL-DW	Schedule for the Evaluation of Individual Quality of Life-Direct Weighting	It is an abridged version of the schedule for evaluation of individual quality of life (SEIQoL). People rate the most significant areas of their lives, assessing satisfaction and the relative importance of these areas to overall quality of life.	• Carolina OC Latorraca *et al*., 2019 [15] • Kassianos AP *et al*., 2017 [34]
SF-36	36-Item Short Form Survey	It is one of the most widely used and easily administered quality-of-life assessments. It includes 36 items, and it is in use in medicare for routine monitoring and evaluation of care outcomes in adult patients.	• Ibeneme SC *et al*., 2019 [39] • Health Quality Ontario. 2016 [8] • Waldemar Siemens *et al*., 2016 [10] • Latorraca COC *et al*., 2017 [25] • Burlacu A *et al*., 2019 [45]
VAS	Visual Analogue Scale	A VAS uses a single-item measurement to assess characteristics or attitudes across a continuum of values. Usually, a VAS gauges the intensity or frequency of diverse symptoms, *e.g*., the extent of pain experienced by patients, from none to severe.	• Waldemar Siemens *et al*., 2016 [10]
WHOQOL-BREF WHOQOL-SRPB	The World Health Organization (WHO) Quality of Life - BREF	The WHOQOL-BREF is a 26-item abridged version of the parent interview assessing four main domains: physical health, psychological health, social relationships, and environment.	• Ibeneme SC *et al*., 2019 [39] • Kun Hyung Kim *et al*., 2016 [11] • Burlacu A *et al*., 2019 [45]
CSS	Client Satisfaction Survey	The CSS is a 10-item tool designed to evaluate satisfaction with the service and the dignity of the treatment.	• Evan T. Hall *et al*., 2019 [26]
EORTC QLQ-C30	European Organization for Research and Treatment of Cancer Quality of Life questionnaire	It is a 9 multi-item self-administered scale that measures functioning across five subscales (physical, role, cognitive, emotional, and social functioning), the impact of symptoms through three subscales (fatigue, pain, and nausea or vomiting), and, finally, the global quality of life. It is often supplemented with other diagnosis-specific questionnaires.	• Evan T. Hall *et al*., 2019 [26] • Kassianos AP *et al*., 2017 [34] • Tobberup R *et al*., 2019 [27] • Sowerbutts AM *et al*., 2018 [22]
FACIT-SP	Functional Assessment of Chronic Illness Therapy-Spiritual	The FACIT-SP presents the patients with a semi-structured interview to assess the presence of thoughts related to death (rated as absent, sub-threshold, or present) from the point of view of spirituality.	• Kassianos AP *et al*., 2017 [34]
Fact-L	Functional Assessment of Cancer Therapy Lung	It is a lung cancer-tailored version of the Functional Assessment of Cancer Therapy - General (FACT-G). Across 36 items, the FACT-L explores five distinct areas (physical, social, family, emotional, and functional well-being).	• Kassianos AP *et al*., 2017 [34]
QUAL-E	Quality of life and quality of care at the end of life	The QUAL-E is a 25-item instrument rating the quality and effectiveness of interventions aimed at enhancing end-of-life care. It encompasses four domains: life completion, symptoms impact, relationship with healthcare providers, and preparation for the end of life.	• Kassianos AP *et al*., 2017 [34] • Fulton JJ *et al*., 2018 [43]
SWLS	Satisfaction With Life Scale	It is a 5-item abridged version of an initial 48-item version, after extensive factorial analysis and retainment of only the cognitive component of well-being. It is used to assess life satisfaction in end-stage chronic conditions.	• Burlacu A *et al*., 2019 [45]
EORTC QLQ-C15 PAL	European Organisation for Research and Treatment of Cancer Quality of Life Core 15 palliative questionnaire	It is a 15-item questionnaire specifically designed for patients in palliative care, typically utilized alongside modules or scales targeting specific disease-related topics.	• Tobberup R *et al*., 2019 [27]
HQLI-R	Hospice Quality of Life Index	It is a concise and straightforward 5-item scale designed for the assessment of treatment outcomes in cancer patients. Its derived indexes assess three areas: psychophysiological, functional, and social/spiritual well-being.	• Warth M *et al*., 2019 [41]

## Data Availability

There was no dataset. All data were in the tables of the article.

## LIST OF ABBREVIATIONS

QoL: Quality of Life
AMSTAR: A Measurement Tool to Assess Systematic Reviews
WHOQOL: World Health Organization Quality of Life
WHO: World Health Organization
HRQoL: Health-Related Quality of Life
PRISMA: Preferred Reporting Items for Systematic Reviews and Meta-Analyses
WHOQOL: World Health Organization Quality of Life
N/A: Not available
Cs: Cannot say
EORTC QLQ: European Organization for Research and Treatment of Cancer Quality of Life questionnaire
EORTC QLQ-C30: European Organization for Research and Treatment of Cancer Quality of Life questionnaire version C - 30 items
ESAS: Edmonton Symptom Assessment Scale
EORTC QLQ-LC13: European Organization for Research and Treatment of Cancer Quality of Life Questionnaire Lung Cancer Module - 13 items
EUROQOL EQ-5D: European Quality of Life Five-Dimensions Questionnaire
FACT-BP: Functional Assessment of Cancer Therapy - Bone Pain
FACT – G: Functional Assessment of Cancer Therapy – General
FACT-G7: Functional Assessment of Cancer Therapy - General - 7 items
HADS: Hospital anxiety and depression scale
IDS-SR30: Inventory of Depressive Symptomatology - Self-rated version - 30 items
IDS-C: Inventory of Depressive Symptomatology - Clinician-rated version
KDQOL: Kidney Disease Quality of Life Instrument
KDQOL SF: Kidney Disease Quality of Life Instrument - Self-rated version
MOS SF-36: Medical Outcomes Study short-form - 36 items
MQOL: McGill Quality of Life Questionnaire
MOS-HIV survey: Medical Outcomes Survey - Human Immunodeficiency Virus survey
NEST: Needs at the End of Life Screening Tool
PedsQL 4.0: Pediatric Quality of Life Inventory - Version 4.0
QOLLTI-F: Quality of Life in Life-Threatening Illness – Family Caregiver Questionnaire
SWB: Subjective well-being measurement
SCID: Structured Clinical Interview for Diagnostic and Statistical Manual of Mental Disorders
SEiQOL-DW: Schedule for the Evaluation of Individual Quality of Life-Direct Weighting
SF-36: 36-Item Short Form Survey
VAS: Visual Analogue Scale
WHOQOL-BREF: The World Health Organization (WHO) Quality of Life - Brief Version
WHOQOL-SRPB: WHOQOL spirituality, religiousness, and personal beliefs (‎SRPB)‎ field-test instrument
CSS: Client Satisfaction Survey
FACIT-SP: Functional Assessment of Chronic Illness Therapy—Spiritual well-being scale
FACT-L: Functional Assessment of Cancer Therapy – Lung
QUAL-E: Quality of Life and Quality of Care at the end of Life
SWLS: Satisfaction With Life Scale
EORTC QLQ-C15 PAL: European Organization for Research and Treatment of Cancer Quality of Life Core 15 items palliative questionnaire
HQLI-R: Hospice Quality of Life Index – Revised
AIDS: Acquired Immune Deficiency Syndrome
RFA: Radiofrequency Ablation

## STANDARDS OF REPORTING

PRISMA guidelines and methodology were followed.

## SUPPLEMENTARY MATERIAL

PRISMA checklist is available as supplementary material on the publisher’s website along with the published article. Supplementary material is available on the publisher’s website along with the published article.

## References

[1] (2002). National cancer control programmes : Policies and managerial guidelines,. World Health Organization.

[2] Page M.J., McKenzie J.E., Bossuyt P.M., Boutron I., Hoffmann T.C., Mulrow C.D., Shamseer L., Tetzlaff J.M., Akl E.A., Brennan S.E., Chou R., Glanville J., Grimshaw J.M., Hróbjartsson A., Lalu M.M., Li T., Loder E.W., Mayo-Wilson E., McDonald S., McGuinness L.A., Stewart L.A., Thomas J., Tricco A.C., Welch V.A., Whiting P., Moher D. (2021). The PRISMA 2020 statement: An updated guideline for reporting systematic reviews.. BMJ.

[3] Shea B.J., Grimshaw J.M., Wells G.A., Boers M., Andersson N., Hamel C., Porter A.C., Tugwell P., Moher D., Bouter L.M. (2007). Development of AMSTAR: A measurement tool to assess the methodological quality of systematic reviews.. BMC Med. Res. Methodol..

[4] Shea B.J., Hamel C., Wells G.A., Bouter L.M., Kristjansson E., Grimshaw J., Henry D.A., Boers M. (2009). AMSTAR is a reliable and valid measurement tool to assess the methodological quality of systematic reviews.. J. Clin. Epidemiol..

[5] Salakari M.R.J., Surakka T., Nurminen R., Pylkkänen L. (2015). Effects of rehabilitation among patients with advances cancer: A systematic review.. Acta Oncol..

[6] McCaffrey N., Bradley S., Ratcliffe J., Currow D.C. (2016). What aspects of quality of life are important from palliative care patients’ perspectives? A systematic review of qualitative research.. J. Pain Symptom Manage..

[7] Maharaj S., Harding R. (2016). The needs, models of care, interventions and outcomes of palliative care in the Caribbean: A systematic review of the evidence.. BMC Palliat. Care.

[8] Health Quality Ontario (2016). Vertebral augmentation involving vertebroplasty or kyphoplasty for cancer-related vertebral compression fractures: A systematic review.. Ont. Health Technol. Assess. Ser..

[9] Barnes H, McDonald J, Smallwood N, Manser R (2016). Opioids for the palliation of refractory breathlessness in adults with advanced disease and terminal illness.. Cochrane Database Syst Rev..

[10] Siemens W., Xander C., Meerpohl J.J., Buroh S., Antes G., Schwarzer G., Becker G. (2016). Pharmacological interventions for pruritus in adult palliative care patients.. Cochrane Libr..

[11] Kim K.H., Lee M.S., Kim T.H., Kang J.W., Choi T.Y., Lee J.D. (2016). Acupuncture and related interventions for symptoms of chronic kidney disease.. Cochrane Libr..

[12] Mochamat C.H., Cuhls H., Marinova M., Kaasa S., Stieber C., Conrad R., Radbruch L., Mücke M. (2017). A systematic review on the role of vitamins, minerals, proteins, and other supplements for the treatment of cachexia in cancer: A european palliative care research centre cachexia project.. J. Cachexia Sarcopenia Muscle.

[13] Schuurhuizen C.S.E.W., Braamse A.M.J., Konings I.R.H.M., Sprangers M.A.G., Ket J.C.F., Dekker J., Verheul H.M.W. (2017). Does severe toxicity affect global quality of life in patients with metastatic colorectal cancer during palliative systemic treatment? A systematic review.. Ann. Oncol..

[14] Janmaat V.T., Steyerberg E.W., van der Gaast A., Mathijssen R.H.J., Bruno M.J., Peppelenbosch M.P., Kuipers E.J., Spaander M.C.W. (2017). Palliative chemotherapy and targeted therapies for esophageal and gastroesophageal junction cancer.. Cochrane Libr..

[15] Latorraca C.O.C., Martimbianco A.L.C., Pachito D.V., Torloni M.R., Pacheco R.L., Pereira J.G., Riera R. (2019). Palliative care interventions for people with multiple sclerosis.. Cochrane Libr..

[16] Dittus K.L., Gramling R.E., Ades P.A. (2017). Exercise interventions for individuals with advanced cancer: A systematic review.. Prev. Med..

[17] Grossman C.H., Brooker J., Michael N., Kissane D. (2018). Death anxiety interventions in patients with advanced cancer: A systematic review.. Palliat. Med..

[18] van Roij J., Fransen H., van de Poll-Franse L., Zijlstra M., Raijmakers N. (2018). Measuring health-related quality of life in patients with advanced cancer: A systematic review of self-administered measurement instruments.. Qual. Life Res..

[19] Rosian K., Hawlik K., Piso B. (2018). Efficacy assessment of radiofrequency ablation as a palliative pain treatment in patients with painful metastatic spinal lesions: A systematic review.. Pain Physician.

[20] Claassen Y.H.M., van der Valk M.J.M., Breugom A.J., Frouws M.A., Bastiaannet E., Liefers G.J., van de Velde C.J.H., Kapiteijn E. (2018). Survival differences with immediate *versus* delayed chemotherapy for asymptomatic incurable metastatic colorectal cancer.. Cochrane Libr..

[21] Abdel-Rahman O., Elsayed Z., Mohamed H., Eltobgy M. (2018). Radical multimodality therapy for malignant pleural mesothelioma.. Cochrane Libr..

[22] Sowerbutts A.M., Lal S., Sremanakova J., Clamp A., Todd C., Jayson G.C., Teubner A., Raftery A.M., Sutton E.J., Hardy L., Burden S. (2018). Home parenteral nutrition for people with inoperable malignant bowel obstruction.. Cochrane Libr..

[23] Friedel M., Aujoulat I., Dubois A.C., Degryse J.M. (2019). Instruments to measure outcomes in pediatric palliative care: A systematic review.. Pediatrics.

[24] Kietpeerakool C., Rattanakanokchai S., Jampathong N., Srisomboon J., Lumbiganon P. (2019). Management of drainage for malignant ascites in gynaecological cancer.. Cochrane Libr..

[25] Latorraca C.O.C., Martimbianco A.L.C., Pachito D.V., Pacheco R.L., Riera R. (2017). Mindfulness for palliative care patients. Systematic review.. Int. J. Clin. Pract..

[26] Hall E.T., Singhal S., Dickerson J., Gabster B., Wong H., Aslakson R.A., Schapira L., Aslakson R., Ast K., Carroll T., Dzeng E., Frechman E., Goett R., Harrison K., Kaye E., Kotwal A., LeBlanc T.W., Shelly Lo, McKenna K., Nageswaran S., Powell V., Powers J., Rotella J., Ullrich C., Vickey T., Wong S., AAHPM Research Committee Writing Group (2019). Patient-reported outcomes for cancer patients receiving checkpoint inhibitors: Opportunities for palliative care—a systematic review.. J. Pain Symptom Manage..

[27] Tobberup R., Thoresen L., Falkmer U.G., Yilmaz M.K., Solheim T.S., Balstad T.R. (2019). Effects of current parenteral nutrition treatment on health-related quality of life, physical function, nutritional status, survival and adverse events exclusively in patients with advanced cancer: A systematic literature review.. Crit. Rev. Oncol. Hematol..

[28] Kavalieratos D., Corbelli J., Zhang D., Dionne-Odom J.N., Ernecoff N.C., Hanmer J., Hoydich Z.P., Ikejiani D.Z., Klein-Fedyshin M., Zimmermann C., Morton S.C., Arnold R.M., Heller L., Schenker Y. (2016). Association between palliative care and patient and caregiver outcomes.. JAMA.

[29] Lau C.H.Y., Wu X., Chung V.C.H., Liu X., Hui E.P., Cramer H., Lauche R., Wong S.Y.S., Lau A.Y.L., Sit R.W.S., Ziea E.T.C., Ng B.F.L., Wu J.C.Y. (2016). Acupuncture and related therapies for symptom management in palliative cancer care.. Medicine.

[30] Guerrero-Torrelles M., Monforte-Royo C., Rodríguez-Prat A., Porta-Sales J., Balaguer A. (2017). Understanding meaning in life interventions in patients with advanced disease: A systematic review and realist synthesis.. Palliat. Med..

[31] Gaertner J., Siemens W., Meerpohl J.J., Antes G., Meffert C., Xander C., Stock S., Mueller D., Schwarzer G., Becker G. (2017). Effect of specialist palliative care services on quality of life in adults with advanced incurable illness in hospital, hospice, or community settings: systematic review and meta-analysis.. BMJ.

[32] Diop M.S., Rudolph J.L., Zimmerman K.M., Richter M.A., Skarf L.M. (2017). Palliative care interventions for patients with heart failure: A systematic review and meta-analysis.. J. Palliat. Med..

[33] Wang C.W., Chow A.Y.M., Chan C.L.W. (2017). The effects of life review interventions on spiritual well-being, psychological distress, and quality of life in patients with terminal or advanced cancer: A systematic review and meta-analysis of randomized controlled trials.. Palliat. Med..

[34] Kassianos A.P., Ioannou M., Koutsantoni M., Charalambous H. (2018). The impact of specialized palliative care on cancer patients’ health-related quality of life: A systematic review and meta-analysis.. Support. Care Cancer.

[35] Schüchen R.H., Mücke M., Marinova M., Kravchenko D., Häuser W., Radbruch L., Conrad R. (2018). Systematic review and meta‐analysis on non‐opioid analgesics in palliative medicine.. J. Cachexia Sarcopenia Muscle.

[36] Gao Y., Wei Y., Yang W., Jiang L., Li X., Ding J., Ding G. (2019). The effectiveness of music therapy for terminally ill patients: A meta-analysis and systematic review.. J. Pain Symptom Manage..

[37] Fulton J.J., LeBlanc T.W., Cutson T.M., Porter Starr K.N., Kamal A., Ramos K., Freiermuth C.E., McDuffie J.R., Kosinski A., Adam S., Nagi A., Williams J.W. (2019). Integrated outpatient palliative care for patients with advanced cancer: A systematic review and meta-analysis.. Palliat. Med..

[38] Cui X., Dong W., Zheng H., Li H. (2019). Collaborative care intervention for patients with chronic heart failure.. Medicine.

[39] Ibeneme S.C., Irem F.O., Iloanusi N.I., Ezuma A.D., Ezenwankwo F.E., Okere P.C., Nnamani A.O., Ezeofor S.N., Dim N.R., Fortwengel G. (2019). Impact of physical exercises on immune function, bone mineral density, and quality of life in people living with HIV/AIDS: A systematic review with meta-analysis.. BMC Infect. Dis..

[40] Lu F., Dong J., Tang Y., Huang H., Liu H., Song L., Zhang K. (2018). Bilateral *vs*. unilateral endoscopic ultrasound-guided celiac plexus neurolysis for abdominal pain management in patients with pancreatic malignancy: A systematic review and meta-analysis.. Support. Care Cancer.

[41] Warth M., Kessler J., Koehler F., Aguilar-Raab C., Bardenheuer H.J., Ditzen B. (2019). Brief psychosocial interventions improve quality of life of patients receiving palliative care: A systematic review and meta-analysis.. Palliat. Med..

[42] Zhou K., Mao Y. (2019). Palliative care in heart failure.. Herz.

[43] Fulton J.J., Newins A.R., Porter L.S., Ramos K. (2018). Psychotherapy targeting depression and anxiety for use in palliative care: A meta-analysis.. J. Palliat. Med..

[44] Burlacu A., Artene B., Nistor I., Buju S., Jugrin D., Mavrichi I., Covic A. (2019). Religiosity, spirituality and quality of life of dialysis patients: A systematic review.. Int. Urol. Nephrol..

